# Efficacy and Safety of Apatinib in the Treatment of Chemotherapy-Refractory Metastatic Colorectal Cancer: A Systematic Review

**DOI:** 10.7759/cureus.29920

**Published:** 2022-10-04

**Authors:** Aujala Irfan Khan, Ghadi D Mashat, Mohammad Hazique, Kokab Irfan Khan, Prasana Ramesh, Suthasenthuran Kanagalingam, FNU Zargham Ul Haq, Nishok Victory Srinivasan, Safeera Khan

**Affiliations:** 1 Research, California Institute of Behavioral Neurosciences & Psychology, Fairfield, USA; 2 Pediatrics, California Institute of Behavioral Neurosciences & Psychology, Fairfield, USA; 3 Internal Medicine, California Institute of Behavioral Neurosciences & Psychology, Fairfield, USA; 4 General Surgery, California Institute of Behavioral Neurosciences & Psychology, Fairfield, USA

**Keywords:** tyrosine kinase inhibitor, safety, efficacy, metastatic colorectal cancer, apatinib

## Abstract

The purpose of this study was to systematically review the current evidence on apatinib and offer a better understanding of its safety and efficacy in metastatic colorectal cancer (mCRC) patients who have not responded to standard chemotherapies. This systematic review was conducted using research from the last 10 years (May 30, 2012, to May 30, 2022) and was obtained from the following databases: PubMed, PubMed Central (PMC), ScienceDirect, and Google Scholar. After removing duplicates, screening titles and abstracts, and applying eligibility criteria and quality appraisal, 11 articles were left for this systematic review (one meta-analysis, eight non-randomized studies, and two traditional reviews). Out of the 11 studies, six were on apatinib monotherapy, while three were on apatinib combination therapy. Apatinib has demonstrated efficacy in the monotherapy and combination therapy trials and has exhibited an acceptable safety profile as the adverse events were predominantly graded 1-2 and could be easily managed. Therefore, apatinib is an encouraging candidate for third-line therapy in chemotherapy-refractory mCRC patients. This conclusion should be confirmed and validated by studies with larger, randomized clinical trials to gain better insight and to directly compare the efficacy and safety of apatinib with all current third-line therapies together so that clinicians can easily assess which treatment modality is superior for chemotherapy-refractory mCRC patients.

## Introduction and background

Colorectal cancer (CRC) is the third most common cancer in the United States and is the second most deadly cancer [1]. Despite dramatic declines in older ages, early-onset CRC (≤ 50 years old) is on the rise in the United States, Australia, and Canada, as well as six other high-income countries [2]. In 2020, there were approximately 1.9 million new CRC cases and 935,000 deaths worldwide, accounting for one in every 10 cancer cases and death [1].

The main treatments available for patients in early or locally advanced stages of CRC are surgery, radiation therapy, and chemotherapy [3]. However, 25% of newly diagnosed CRC patients will have metastases at diagnosis [4]. Patients with CRC have a five-year survival rate that ranges from 90% in the localized stages to 14% in the advanced stages [5]. The median overall survival (OS) is approximately 30 months in clinical trial populations and around two years in the general community [6].

The first- and second-line therapy for metastatic colorectal cancer (mCRC) consists of fluorouracil-based doublet regimens (5-fluorouracil/leucovorin plus oxaliplatin and 5-fluorouracil/leucovorin plus irinotecan) and biologic-targeted therapies including either endothelial growth factor receptor (cetuximab or panitumumab) targeted treatment or vascular endothelial growth factor (VEGF; bevacizumab), or programmed cell death protein 1 (PD-1) inhibitors [7-8].

After two or more lines of systemic treatment, most mCRC patients experience disease progression [9]. Nonetheless, a significant proportion can maintain a markedly better Eastern Cooperative Oncology Group performance status (ECOG PS) after previous chemotherapy, encouraging them to undertake further therapy [9]. The National Comprehensive Cancer Network (NCCN) guidelines recommend regorafenib or TAS-102 (combination of trifluridine and tipiracil hydrochloride) for third-line treatment of mCRC [10]. In China, fruquintinib is also approved in addition to the drugs mentioned above for third-line treatment [11]. Regorafenib, fruquintinib, and TAS-102 have superior efficacy compared with placebo, making them appropriate for third-line therapy, but their survival effect in phase III trials is modest [12-15]. Moreover, third-line or further-line treatment can increase OS expectancy in mCRC patients [16]. As a result, the therapy options offered are insufficient to address existing medical needs [17].

Angiogenesis, primarily controlled by the VEGF pathway, plays a crucial role in the progression of CRC, and anti-angiogenesis therapy has been a vital treatment option in mCRC [18]. Anti-VEGF antibody bevacizumab, for example, has been utilized to treat mCRC in both first- and second-line settings in combination with standard chemotherapy [19-20]. Another example is the small-molecule multi-receptor tyrosine kinase inhibitors (regorafenib and fruquintinib) indicated as third-line therapy [12-14]. However, they only provide moderate benefits and cause adverse events (AEs) that are difficult to manage [12-14].

Apatinib mesylate, also known as YN968D1 and rivoceranib, is a novel anti-angiogenic oral small molecule drug that inhibits VEGF receptor-2 (VEGFR-2) and the tyrosine kinases: cellular sarcoma (c-SRC) and type III receptor tyrosine kinase (c-Kit) [21-23]. In China, apatinib was licensed and released in 2014 as a subsequent line of treatment for advanced gastric cancer [24]. Also, many clinical studies have recently demonstrated its effectiveness in malignancies such as non-small cell lung cancer, breast cancer, and hepatocellular carcinoma [25-28]. Apatinib has demonstrated good anti-tumor activity both in vitro and in vivo in CRC, according to Mi et al. [29].

This systematic review will focus on the efficacy and safety of apatinib for the treatment of mCRC in patients who have not responded to standard chemotherapies.

## Review

Methods

Preferred Reporting Items for Systematic Reviews and Meta-Analyses (PRISMA) 2020 guidelines were used for this systematic review [30].

Databases and Keywords Used

This systematic review was conducted using research from the following databases: PubMed, PubMed Central (PMC), ScienceDirect, and Google Scholar.

Medical Subject Headings (MeSH) keywords used were: ( "Colorectal Neoplasms/analysis"[Majr] OR "Colorectal Neoplasms/complications"[Majr] OR "Colorectal Neoplasms/drug therapy"[Majr] OR "Colorectal Neoplasms/metabolism"[Majr] OR "Colorectal Neoplasms/mortality"[Majr] OR "Colorectal Neoplasms/pathology"[Majr] OR "Colorectal Neoplasms/physiology"[Majr] OR "Colorectal Neoplasms/physiopathology"[Majr] OR "Colorectal Neoplasms/prevention and control"[Majr] OR "Colorectal Neoplasms/therapy"[Majr] ) AND "apatinib" [Supplementary Concept]

Keywords used for ScienceDirect and Google Scholar were “Colorectal cancer” and “Apatinib.”

Inclusion Criteria

All studies were assessed based on the following inclusion criteria: (a) papers from the past 10 years (May 30, 2012, to May 30, 2022), (b) papers published in the English language, (c) papers focusing on the adult population aged ≥ 18 years, (d) papers relevant to the question, and (e) full-text papers.

Exclusion Criteria

Studies were removed based on the following exclusion criteria: (a) papers discussing the pediatric population, (b) unpublished literature, (c) grey literature, and (d) abstract-only papers.

Study Screening and Quality Appraisal

The first and second authors separately performed the title, abstract, and full-text screening. Any disagreements were settled by the third author at any juncture. After the final papers for this review were chosen, all of the authors named were equally active in evaluating each one for quality and gathering data for this systematic review. We compared and contrasted the data from each chosen study and retrieved information about apatinib's effectiveness (parameters assessed were progression-free survival [PFS], OS, objective response rate [ORR], and disease control rate [DCR]), and safety (any AEs).

Newcastle-Ottawa scale: A score of 0-3 is a high risk, 4-6 is moderate risk, and 7-9 is low risk. All high-risk studies were excluded, and only moderate and low-risk studies were included.

Assessing the Methodological Quality of Systematic Reviews (AMSTAR) checklist: All articles satisfying ≥70% of the checklist questions were included.

Scale for the Assessment of Narrative Review Articles (SANRA) checklist: All articles satisfying ≥70% of the checklist questions were included.

The tools mentioned in Table 1 were used for the relevant study type.

**Table 1:** Study type and the relevant quality assessment tool used AMSTAR, Assessing the Methodological Quality of Systematic Reviews; SANRA, Scale for the Assessment of Narrative Review

Type of study	Quality assessment tool used	Number of studies
Meta-analysis	AMSTAR Checklist	1
Non-randomized studies	Newcastle-Ottawa scale	8
Research papers without clear methods section	SANRA checklist	2

Results

**Figure 1:**
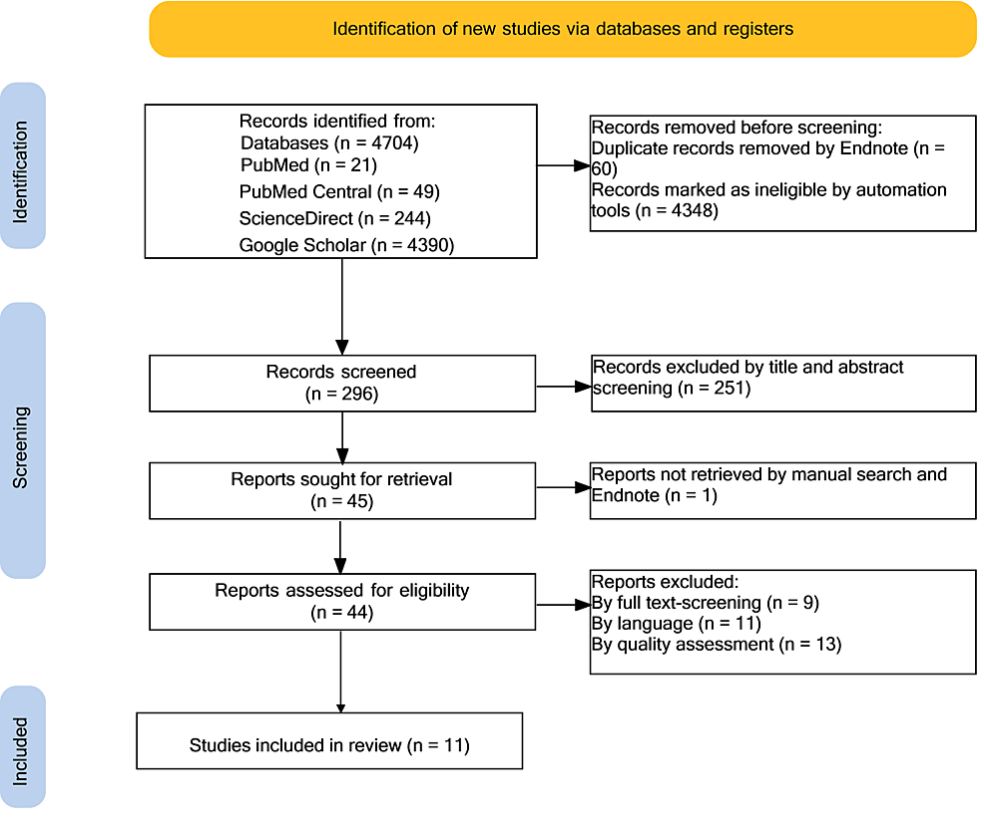
PRISMA flowchart PRISMA, Preferred Reporting Items for Systematic Reviews and Meta-Analyses; n, number of records

A total of 4,704 results were identified from the databases mentioned above, and 4,408 results were removed because of duplicates (60 results) and automation tools (4348 results). A further 251 were excluded by screening titles and abstracts. The remaining 45 were sought for retrieval, but one could not be retrieved. In total, 44 reports were evaluated for eligibility, and 33 were removed after screening for full text, English language only papers, and quality assessment. Eleven articles were left for use in this systematic review (one meta-analysis, eight non-randomized studies, and two traditional reviews). The PRISMA flowchart in Figure 1 demonstrates the filtering process.

Table 2 briefly summarizes the studies included in this systematic review.

**Table 2:** Brief overview of the studies mentioned in this systematic review PD-1, programmed cell death protein 1; CRC, colorectal cancer; S-1, a combination of tegafur, gimeracil, and oteracil; mCRC, metastatic colorectal cancer; KDR, kinase insert domain receptor; NA, not assessed; PFS, progression-free survival

Author and year of publication	Type of study	Purpose of the study	Number of patients/studies	Conclusion
Pan et al., 2022 [31]	Prospective	To assess the effect and safety of PD-1 inhibitor + apatinib vs. apatinib monotherapy	45	PD-1 inhibitor + apatinib, compared to apatinib monotherapy, can improve efficacy and survival while maintaining tolerable safety in advanced CRC patients
Dai et al., 2022 [11]	Retrospective	To assess the effectiveness and safety of apatinib + S-1 vs. regorafenib vs. fruquintinib in refractory mCRC	114	Treatment with low dose apatinib + S-1 was promising when compared to regorafenib and fruquintinib
Bai et al., 2021 [32]	Retrospective	To evaluate the effect of KDR genetic variation on apatinib therapy in refractory mCRC	108	Apatinib is more effective in refractory mCRC patients with rs2071559 polymorphism
Li et al., 2021 [16]	Single-arm, prospective, multicenter, phase II trial	To evaluate the effectiveness and safety of low dose apatinib + S-1 in refractory mCRC	29	Low dose apatinib + S-1 is an encouraging candidate for refractory mCRC
Tian et al., 2021 [33]	Traditional review	To evaluate the effectiveness and the relevant response biomarkers of apatinib in different malignancies	NA	Apatinib has a lot of potential in multiple malignancies, and more research is needed for biomarkers
Wang et al., 2020 [9]	Single-arm, prospective, multicenter study	To assess apatinib monotherapy for refractory mCRC	48	Promising efficacy of apatinib monotherapy was seen in refractory mCRC
Shao et al., 2020 [34]	Traditional review	To discuss the safety of apatinib in metastatic cancers	NA	Preventing and treating adverse events will improve the quality of life
Yang et al., 2019 [35]	Single-arm, prospective, single-center study	To assess apatinib monotherapy for refractory mCRC	14	Apatinib monotherapy demonstrated efficacy in refractory mCRC
Liang et al., 2018 [36]	Retrospective	To assess apatinib monotherapy for refractory mCRC	36	Apatinib is a promising candidate, and its effectiveness is not affected by previous bevacizumab treatment
Liao et al., 2018 [17]	Retrospective	To assess apatinib monotherapy for refractory mCRC	53	The apatinib group had longer PFS and better disease control than the observation group
Sun et al., 2018 [37]	Meta-analysis	To evaluate the effectiveness and safety of apatinib in refractory malignancies	21 studies	Better disease control is seen in patients treated with apatinib. Most adverse events are manageable and well-tolerated.

Discussion

Efficacy endpoints are PFS (defined as the duration from initiation of intervention to disease progression or death from any cause, whichever occurred first), OS (defined as the duration from the onset of treatment until death from any cause), ORR (defined as the proportion of chemotherapy-refractory mCRC patients with partial or complete response to the intervention), and DCR (defined as the proportion of chemotherapy-refractory mCRC patients with complete response, partial response, or stable disease after treatment initiation).

Efficacy of Apatinib Monotherapy

Wang et al. conducted a single-arm, prospective, multicenter study to investigate the efficacy of apatinib monotherapy in refractory mCRC [9]. All 48 enrolled patients received apatinib monotherapy [9]. Median PFS was 4.8 months (95% confidence interval [CI]: 3.653-5.887), and median OS was 9.1 months (95% CI: 5.155-13.045) [9]. ORR was 8.3%, and DCR was 68.8% [9]. Results were unaffected by prior anti-angiogenic treatments [9].

Similarly, in a single-arm, prospective, single-center study by Yang et al., all 14 patients received apatinib, resulting in a median PFS of six months, median OS of 10 months, ORR of 28.57%, and DCR of 71.43% [35]. A comparable result was seen in a retrospective study by Liang et al., which had 36 mCRC patients treated with apatinib monotherapy as third-line treatment [36]. It demonstrated a median PFS of 4.8 months, median OS of 10.1 months, ORR of 11.1%, and DCR of 77.8% [36]. Moreover, the history of bevacizumab use did not affect median PFS and median OS [36].

The retrospective study by Liao et al. included 53 patients, of which 27 were given apatinib, and 26 with similar clinical characteristics were subject to observation [17]. Median PFS (2 vs. 1.1 months; p=0.001) was improved in the apatinib group versus the observation group [17]. Median OS (five months vs. four months; p=0.722) was increased in the apatinib group versus the observation group [17]. However, no statistical difference was noted; this may change with a larger cohort size [17].

In a retrospective study, Bai et al. evaluated the effect of kinase insert domain receptor (KDR) genetic variation on apatinib monotherapy in 108 mCRC patients [32]. Median PFS was 3.6 months (95% CI: 3.03-4.17 months), and median OS was 8.9 months (95% CI: 7.57-10.23 months) [32]. ORR was 5.6% and DCR was 69.4% [32]. Polymorphism rs2071559 was clinically significant according to the investigation of KDR genetic variation [32].

Sun et al. conducted a meta-analysis to assess the effectiveness and safety of apatinib in refractory malignancies [37]. Out of the 21 studies (735 patients) they evaluated, four were on apatinib in chemotherapy-refractory mCRC (103 patients) [37]. They demonstrated a pooled ORR of 13% and a pooled DCR of 79% in mCRC patients using apatinib monotherapy [37].

Efficacy of Apatinib Combination Therapy

Li et al. directed a single-arm, prospective, multicenter, phase II trial to appraise low-dose apatinib combined with S-1 (combination of tegafur, gimeracil, and oteracil) in refractory mCRC patients [16]. A total of 29 patients were enrolled [16]. All patients enrolled received apatinib and S-1 [16]. Median PFS was 7.9 months, and the median OS was 12.9 months [16]. The ORR was 13.79%, and DCR was 89.77% [16]. Previously targeted therapies did not influence outcomes [16].

The study by Dai et al. was a retrospective cohort to evaluate the effectiveness and safety of apatinib and S-1 versus regorafenib versus fruquintinib in chemotherapy-refractory mCRC patients [11]. The study included 114 patients (apatinib and S-1 group = 43, regorafenib group = 36, and fruquintinib group = 35) [11]. PFS (median PFS was 3.9 months vs. 2.4 months vs. 3.1 months) was significantly improved in the apatinib and S-1 group versus regorafenib group versus fruquintinib group, respectively [11]. Median OS (8.2 months vs. 7.5 months vs. 7.8 months) was slightly increased, but no statistical difference was seen in the apatinib and S-1 group versus regorafenib group versus fruquintinib group [11]. The ORR was 2.3% in the apatinib plus S-1 group and 0% in both the regorafenib and the fruquintinib groups [11]. The DCR (83.7% vs. 66.7% vs. 71.4%) was increased in the apatinib and S-1 group versus the regorafenib group versus the fruquintinib group [11]. Nevertheless, no statistical significance was observed, which could be due to the small sample size [11].

Pan et al. conducted a prospective study to assess the efficacy and safety of a PD-1 inhibitor and apatinib versus apatinib monotherapy on mCRC patients as third-line treatment [31]. Of the 45 patients who participated in this study, 20 were given a PD-1 inhibitor and apatinib, while 25 received apatinib monotherapy [31]. PFS (median PFS was 7.5 months vs. 4.8 months, and the one-year PFS rate was 32.5% vs. 9.9%; p=0.038) and OS (median OS was 12.3 vs. 8.7 months, and the one-year OS rate was 50.7% vs. 27%; p=0.048) were improved in the PD-1 inhibitor plus apatinib group versus the apatinib group, respectively [31]. The ORR (20% vs. 8%; p=0.383) and DCR (70% vs. 52%; p=0.221) were increased in the PD-1 inhibitor plus apatinib group versus the apatinib group, but no statistical significance was found [31]. The small sample size leading to a decreased statistical power could explain the lack of statistical significance observed [31]. Moreover, in the PD-1 inhibitor plus apatinib group, the PD-1 inhibitor used was camrelizumab for some patients and pembrolizumab for others [31]. It is unclear which PD-1 inhibitor was more efficacious and how many received which type [31].

Potential Factors That Can Help Gauge the Response to Apatinib in Chemotherapy-Refractory mCRC

Apatinib monotherapy: Wang et al. found that the occurrence of hand-foot syndrome (HFS) during the first 28 days (p=0.007), low neutrophil-to-lymphocyte ratio (p=0.040), and early decrease in carbohydrate antigen 19-9 (p=0.011) were linked to increased PFS [9]. Additionally, Liao et al. demonstrated that an increased PFS was associated with ≤ 2 metastatic sites, and decreased OS was seen in patients with high ECOG scores, lymph node metastasis, or right-sided colon cancer [17]. Bai et al. showed that rs2071559 polymorphism of the KDR gene was clinically significant (0.22 was the minor allele frequency, and the genotype status was in agreement with Hardy-Weinberg equilibrium; p=0.949) [32]. The genotypes of rs2071559 were identified using the size of polymerase chain reaction bands: TT genotype (one 271 base pairs (bp) band), CC genotype (one 108 bp band and one 163 bp band), and TC genotype (one 271 bp band, one 108 bp band, and one 163 bp band) [32]. Median PFS (4.1 months vs. three months; p=0.012) and median OS (10.5 months vs. 6.1 months; p=0.007) were improved in TT genotype compared to TC/CC genotype [32]. The ORR was 6.1% vs. 4.8% (p=0.774), and DCR was 74.2% versus 61.9% (p=0.175) in TT genotype and TC/CC genotype, respectively [32]. Moreover, multivariate analysis indicated that the following were an independent factor for OS: rs2071559 polymorphism (hazard ratio [HR]=0.65, p=0.021), age (HR=0.82, p=0.040), ECOG score (HR=0.76, p=0.024), and tumor location (HR=1.38, p=0.017) [32].

Apatinib combination therapy: Pan et al. showed that patients with bevacizumab history (p=0.018) or a single metastatic site (p=0.033) had increased OS (but no difference in PFS) in the PD-1 inhibitor plus apatinib group versus the apatinib group [31]. This indicates that in patients with bevacizumab failure or a single metastatic site, PD-1 inhibitor plus apatinib therapy could offer potential benefits [31]. Moreover, Li et al. found that when treating patients with low dose apatinib and S-1, lower neutrophil-to-lymphocyte ratios showed longer median PFS (5.6 vs. 3.5 months, p=0.006) and median OS (19.1 vs. eight months, p=0.005) than those with greater ratios [16]. Also, Li et al. demonstrated that a lower D-dimer was linked to an increased OS when compared to a higher one (15.2 vs. eight months, p=0.033) [16]. Furthermore, Dai et al. found that the following were independent predictors of decreased PFS and OS: age < 70 years (PFS: HR=2.4, p=0.015; OS: HR=2.6, p=0.046), ECOG PS 2 (PFS: HR=2.1, p=0.037; OS: HR=10.9, p=0.000), and fourth-line and above treatment (PFS: HR=2.3, p=0.000; OS: HR=2.7, p=0.002) [11]. Additionally, they discovered that regorafenib use was associated with decreased PFS (HR=2.1, p=0.005), while having more than two metastatic sites was an independent predictor of decreased OS (HR=2.1, p=0.011) [11].

Even though numerous possible prognostic indicators for apatinib have been identified, none have found widespread usage in clinical settings [33]. This is because the quality of current evidence is suboptimal, and the effectiveness of different types of markers in various cancers varies significantly [33]. There needs to be a large sample size and prospective trials focusing on identifying specific indicators [33].

Safety of Apatinib

The safety profile of third-line or further-line mCRC treatments can aid clinicians in making therapy decisions and help prevent and treat possible AEs to improve quality of life [34]. AEs were graded using the common terminology criteria for adverse events (CTCAE) [9,11,16-17,31-32,35-37].

Apatinib monotherapy: According to Sun et al., 84% of patients reported experiencing apatinib's AEs [36]. However, most AEs were in grades 1-2 [9,17,32,35-37]. They could be easily managed and were well tolerated [9,17,32,35-37]. The most common grade 3-4 AEs were proteinuria, HFS, and hypertension [9,17,32,35-37]. Moreover, there were no appreciable differences between the higher-dose and lower-dose groups in the occurrences of the three grade 3-4 AEs that were most often reported (proteinuria, HFS, and hypertension: 6%, 7%, and 4%, respectively) [37].

Apatinib combination therapy: In the study by Li et al., most AEs were grades 1-2 and were well tolerated and controlled, while 10 grade 3 AEs were observed, and the frequency of each grade 3 AE was less than 5% [16]. Additionally, Dai et al. showed that the apatinib plus S-1 group had a greater incidence of hematological toxicity (anemia in 62.8%, neutropenia in 30.2%, and thrombocytopenia in 39.5%), HFS (58.3%) was more predominant in the regorafenib group, and hypertension (45.7%) was substantial in the fruquintinib group [11]. The combination with cytotoxic drugs after multi-line chemotherapy may contribute to the enhanced hematological toxicities [11]. Regorafenib and fruquintinib were inferior to apatinib and S-1 in non-hematological toxicities such as hypertension, proteinuria, and HFS [11]. Furthermore, Pan et al. demonstrated that the majority of the AEs were of grade 1-2, and their incidence was similar between groups, except for the incidence of cutaneous capillary proliferation, which was elevated in the PD-1 inhibitor plus apatinib group compared with the apatinib group (25.5% vs. 0.0%; p=0.013) [31].

Overall, the safety profile of apatinib, whether taken as monotherapy or in combination with other treatments, is acceptable, as the majority of the AEs were graded 1-2 and easily manageable.

Limitations

There are some potential limitations in our systematic review. All studies had a small sample size and were conducted in China, limiting the results' generalizability. Also, most of the studies included were single-center studies, which introduce inherent selection bias in the data. As a result, large-scale, prospective, multicenter randomized clinical trials conducted in different populations are urgently required to investigate whether apatinib is more effective as a monotherapy or combination therapy.

## Conclusions

As far as we know, this is the first systematic review to evaluate the efficacy and safety of apatinib as third-line therapy in advanced CRC patients. Most of the included studies revealed that apatinib has significant efficacy as monotherapy and combination therapy. Moreover, multiple factors identified can help gauge the response to apatinib in monotherapy or combination therapy, but the quality of evidence is low. Furthermore, apatinib has an acceptable safety profile as the AEs were predominantly graded 1-2 and could be easily managed. Therefore, apatinib is an encouraging candidate for third-line therapy in chemotherapy-refractory mCRC patients. This conclusion should be confirmed and validated by studies with larger sample sizes and prospective, multicenter randomized clinical trials worldwide to gain better insight regarding its safety and efficacy. Also, additional clinical trials are required to directly compare the efficacy and safety of apatinib with all current third-line therapies so that clinicians can assess which treatment modality is superior for chemotherapy-refractory mCRC patients.
